# Trains of Epidural DC Stimulation of the Cerebellum Tune Corticomotor Excitability

**DOI:** 10.1155/2013/613197

**Published:** 2013-05-20

**Authors:** Nordeyn Oulad Ben Taib, Mario Manto

**Affiliations:** ^1^Service de Neurochirurgie, CHU Saint-Pierre-ULB, 1070 Bruxelles, Belgium; ^2^FNRS Neurologie, Unité d'Etude du Mouvement, Hôpital Erasme-ULB, 808 Route de Lennik, 1070 Bruxelles, Belgium

## Abstract

We assessed the effects of anodal/cathodal direct current stimulation (DCS) applied epidurally over the cerebellum. We studied the excitability of both the motor cortex and the anterior horn of the spinal cord in adult rats under continuous anesthesia. We also investigated the effects on the spatial representation of a couple of agonist/antagonist muscles on primary motor cortex. Moreover, we evaluated the effects on the afferent inhibition in a paradigm of conditioned corticomotor responses. Anodal DCS of the cerebellum (1) decreased the excitability of the motor cortex, (2) reduced the excitability of *F* waves, as shown by the decrease of both mean *F*/mean *M* ratios and persistence of *F* waves, (3) exerted a “smoothing effect” on corticomotor maps, reshaping the representation of muscles on the motor cortex, and (4) enhanced the afferent inhibition of conditioned motor evoked responses. Cathodal DCS of the cerebellum exerted partially reverse effects. DCS of the cerebellum modulates the excitability of both motor cortex and spinal cord at the level of the anterior horn. This is the first demonstration that cerebellar DCS tunes the shape of corticomotor maps. Our findings provide a novel mechanism by which DCS of the cerebellum exerts a remote neuromodulatory effect upon motor cortex.

## 1. Introduction

The dynamic modulation of the excitability of the motor cortex is critical for motor control. It depends on several elemental parameters: the excitability of single cells, the synaptic strength, and the balance between excitatory cells and inhibitory cells [1, 2]. The cerebellum is one of the subcortical structures modulating the excitability of the motor cortex and the spinal cord [3]. It is presumed that defects in the tuning of the excitability of the corticomotor responses contribute to the sensorimotor learning deficits in cerebellar patients [4]. Since we currently lack efficient therapies in numerous forms of cerebellar disorders encountered during daily practice, there is a need to identify novel strategies that might be used to antagonize cerebellar motor deficits. The mechanisms of these deficits depend on the type of cerebellar damage. Indeed, cerebellar cortex inhibits strongly cerebellar nuclei, which themselves stimulate contralateral motor cortex and ipsilateral anterior horn of the spinal cord. Therefore, cerebellar cortical lesions induce a disinhibition of cerebellar nuclei, which results in an excitatory overdrive along the dentatothalamocortical pathway [5]. When the lesion is extensive and includes cerebellar nuclei, such as in hemicerebellar ablation, an excess of inhibition occurs in the contralateral motor pathways [4].

Recent years have seen a promising explosion in the use of noninvasive electrical or magnetic stimulation methods as research or therapeutic tools. In particular, transcranial (over the skull) DC stimulation (DCS) applied over the cerebellum is a resurging tool currently under investigation to speed up learning of reaching or adaptation during locomotion [6, 7]. The important work of Galea et al. [8] has demonstrated that cathodal transcranial DCS of the cerebellum decreases cerebellum to motor cortex inhibition, in contrast with anodal transcranial DCS. These effects were found to be specific to the cerebellocortical connections with no changes in brainstem excitability measures. The cathodal effect lasted up to 30 min after the cessation of transcranial DCS. As underlined recently, there is a compelling need for animal models to test the effects of DCS and evaluate its implications in complex cerebral processes, because little is known about the cellular mechanisms of the neuromodulatory aftereffects of transcranial DCS [9]. We have shown previously in rats maintained under continuous anesthesia that trains of anodal DCS applied over the motor cortex contralaterally to a hemicerebellar ablation antagonize the decrease in motor cortex excitability, without changing the excitability of the spinal cord as assessed by the *H* reflex or the *F* waves [4]. Continuous or intermittent anodal DCS induces a polarity-dependent site-specific modulation of brain activity [10]. Anodal stimulation of the motor cortex increases cortical excitability below the site of stimulation, not only by reducing intracortical inhibition but also by enhancing facilitation [10, 11]. Anodal DCS causes a depolarization of the neural tissue, inducing a subthreshold membrane potential shift and increasing neural firing rate, therefore, enhancing the overall neural activity of the stimulated area [12].

In order to gain more insights into the mechanisms of action of DCS applied over the cerebellum, we tested the hypothesis that this neurostimulation method modulates the excitability of both the motor cortex and the anterior horn of the spinal cord in anesthetized rats. In particular, we assessed the effects of DCS on corticomotor discharges and corticomotor representation over motor cortex of a couple of agonist-antagonist muscles.

## 2. Materials and Methods

Studies were performed in adult Wistar rats, following approval of the institutional animal care committee of the Free University of Brussels. Procedures to minimize discomfort were used during the experiments. Surgical procedures were conducted by a neurosurgeon familiar with neurosurgery in rodents. All efforts were made to reduce the number of animals undergoing surgery to minimum. Male Wistar rats (weight: 270 to 390 gr; *n* = 13) were anesthetized using an intraperitoneal administration of chloral hydrate (400 mg/kg i.p.) [13, 14]. Chloral hydrate was subsequently administered continuously using the i.p. route at a flow rate of 2 *μ*L/min (CMA micropump, CMA, Sweden). Rats were thus maintained anesthetized. This continuous infusion is required to obtain stable baseline corticomotor responses and reproducible results. Anesthesia depth was adjusted for absence of abdominal contractions in response to tail pinch. Rats were fixed in a stereotaxic apparatus (Kaps, Germany). Head was levelled and secured by ear bars and a tooth holder [15]. The scalp was shaved, cut sagitally, and tissues overlying the cranium were removed [14, 16]. The dura over the cerebellum was carefully exposed under a surgical microscope.

### 2.1. Corticomotor Responses and Mapping Procedure

We investigated the corticomotor responses evoked in the left/right gastrocnemius muscle and left/right tibialis anterior muscle following stimulation of the right/left motor cortex, before (basal condition) and after application of trains of anodal or cathodal DCS over left cerebellar hemisphere (see below). For both the gastrocnemius muscle and the tibialis anterior muscle, motor maps were obtained by applying a matrix of stimulation of 6 × 9 sites (6 sites along the sagittal axis and 9 sites along the coronal axis). We used a successive point-by-point stimulation method along the sagittal and coronal axes with monophasic rectangular pulses. We extracted the peak-to-peak amplitudes of the corticomotor responses and represented the corticomotor maps of amplitudes of responses using contour plots. The goal was to characterize the motor representation of each muscle [4]. The impedance was kept below 5 KOhms. The technique to identify the precise location of the “hot spot” corresponding to the largest motor evoked potential (MEP; confirmed by epidural stimulation with tungsten microelectrodes TM33A05, World Precision Instruments, UK) was reported earlier [4]. In order to quantify the “focusing effect” exerted by anodal DCS (ADCS) on corticomotor maps (see Section 3), we counted the number of peaks of MEP with values above 50% of maximal amplitude of MEP in the basal condition (NP50; according to a linear scale along the *z*-axis) in the 3 experimental conditions (baseline, after ADCS and after cathodal DCS—CDCS) for both the left gastrocnemius muscle and the left tibialis anterior muscle.

For the assessment of the recruitment curves of corticomotor responses related to the gastrocnemius muscles (tibialis anterior muscles), we used the location of 3 (2) mm laterally and 0.5 mm posterior to bregma for stimulation of the motor cortex [4]. This location is consistent with stimulation sites used in other studies [1, 16]. We applied a detection of motor threshold (MT) defined as the lowest intensity eliciting at least 5 out of 10 evoked responses with an amplitude >20 microVolts. We increased the intensity of stimulation with steps of 0.1 mAmp until maximum. Corticomotor responses were analyzed to confirm the classical sigmoid course using a sigmoid fitting with 3 parameters: *y* = *a*/(1 + exp⁡(−(*x* − *x*_0_)/*b*)) [1, 17]. We also computed the rising slope of the straight line from the first point to the sixth point of the recruitment curve (slope_1–6_). Subsequently, motor cortex was stimulated at an intensity of 130% of MT to assess latencies and amplitudes of corticomotor potentials [17]. Peak-to-peak amplitudes in responses of the left (right) gastrocnemius muscle were studied for sets of 10 corticomotor responses. We used subcutaneous electrodes (Technomed 017K025) implanted in muscles. We obtained similar results by folding wires (Wire silver, AGT0510, World Precision Instruments) into flat plates which were implanted into a subcutaneous pocket over the gastrocnemius muscle (see also [1]). To record compound muscle action potentials (CMAPs), electrical stimuli were applied (needle electrodes) at the level of the ipsilateral sciatic nerve at about 16 mm laterally from midline [3, 17]. CMAPs in the left/right gastrocnemius muscles were obtained using electrical stimuli (duration: 1 msec; square waves) delivered by a stimulation unit (NeuroMax 4, Xltek, Canada).

### 2.2. Afferent Inhibition

The site of stimulation of the sciatic nerve described above was used for conditioning corticomotor responses [14]. The intensity of stimulation was selected when the stimulus elicited a very slight twitch of the hindlimb. Test stimulus on the motor cortex was preceded by a conditioning stimulus (DS70 stimulator, Digitimer, UK) in the contralateral nerve at an interstimulus interval of 45 msec [14]. We computed the ratios of the amplitudes of MEPs obtained with conditioning divided by amplitudes of unconditioned MEPs.

### 2.3. *H* Reflex, *F* Waves, and *M* Responses

We studied the *H* reflex (evoked by electrical stimulation of nerves and which represents an electrical analogue of the monosynaptic stretch reflex), the *F* wave (whose intensity is proportional to the excitability of the anterior corn in spinal cord), and the direct motor responses (*M* responses, which are generated by motor axons activated directly by the electrical stimulus applied on the nerve) [18]. The *F* waves and *M* responses were studied in the left/right plantaris muscle using a method adapted from Gozariu et al. [19]. Electrical stimulation of the left/right tibial nerve was performed using needle electrodes inserted subcutaneously at the ankle, behind the medial malleolus. Electrical stimuli consisted of single square-wave shocks of 0.5 msec duration, delivered every 6 seconds. EMG recordings were made from the ipsilateral plantaris muscle through a pair of needle electrodes inserted in the distal third of the sole (filters: 30 Hz–1.5 KHz). Integrals of *H* and *M* responses were plotted against stimulus intensity to analyze the recruitment curves for the *H*/*M* ratios. In particular, we studied the *H*/*M*_max⁡_ ratios at 2 times and 3 times *M* thresholds because they represent robust indices of the excitability of the monosynaptic reflexes [3, 13, 17]. We also assessed the *F* wave persistence (percentage of *F* waves present in a series of stimuli) and the ratio mean *F*/mean *M* wave amplitudes following 50 supramaximal stimuli. These studies were performed in the basal condition and were repeated after anodal or cathodal DCS.

### 2.4. DCS

After basal measurements, trains of electrical stimuli were applied over the cerebellum with the anode (or cathode) placed at the level of the left hemisphere in front of Crus I/Crus II [4, 20]. The cathode (the anode; low impedance metallic electrode with a diameter of 0.8 mm, see [4]) was fixed 5 mm anteriorly to the bregma in right supraorbital region, inserted epicranially. For anodal stimulation, we used a method similar to the one described by Fregni et al. [21]. A small plastic jacket was fixed over left cerebellar hemisphere with dental cement and filled with saline solution (0.9% NaCl) to obtain a contact area of 7.1 mm². For cathodal stimulation, the cathode was applied over the cerebellar hemisphere. Duration of stimulation was 20 minutes (this duration of stimulation has been used in other studies (see [12]); a duration of stimulation of 7 minutes at 1 mA is known to induce significant changes of motor cortical excitability in human). Stimulus intensity was 0.4 mAmp (current supplied by a constant current A310–A365 stimulator; a battery charger A362 was used to charge the stimulator between the experiments—World Precision Instruments, UK), an intensity used in other rat studies [22]. DCS was applied directly onto the dura to ensure a defined contact area over the cerebellar cortex [22]. We previously tested the effects of the duration of pulses on motor cortex excitability [4]. We found that the highest effects on motor cortex excitability were observed with the longest pulses tested (10-second pulses delivered every 11 sec: ~0.091 Hz), which were selected. The current density was 5.12 mAmp/cm² (as compared to 2.86 mAmp/cm² in the study of Fregni et al., 2007 [21]).

In a preliminary study, we assessed the duration of the aftereffect after anodal and cathodal stimulation of the left cerebellar hemisphere in 3 rats. We observed that changes in corticomotor excitability lasted between 55 and 65 minutes, whether anodal or cathodal stimulation was applied first. Values of corticomotor excitability returned to baseline after this period. Therefore, we used a timing of 80 minutes between measurements related to anodal or cathodal DCS. In addition, anodal or cathodal DCS was administered randomly after the first set of recordings corresponding to basal values. Therefore, we performed the following successive sets of measurements for both parameters of motor cortex excitability and spinal cord excitability in a total of 13 rats:

- basal values, post-DCS anodal (post-ADCS), and post-DCS cathodal (post-CDCS) (in *n* = 6 rats);
- basal values, post-CDCS, and post-ADCS (in *n* = 7 rats).

We also applied a random selection of side (left versus right side for EMG measurements) for recordings of corticomotor responses and parameters of spinal cord excitability.

### 2.5. Verification of Absence of Cerebellar Lesion Induced by DCS

At the end of the experiments, brains were extracted and carefully inspected under a microscope to evaluate a possible lesion of the cerebellar cortex, after administration of an overdose of chloral hydrate (1000 mg/kg i.p.) and decapitation. We inspected specifically the cerebellar, motor, and premotor areas for possible lesions related to the experiments. Previous studies with similar intensities and durations of stimulation were not associated with electrically induced lesions [4].

### 2.6. Statistical Analysis

Normality of data was assessed using the Kolmogorov-Smirnov test (Sigma Stat Software, Jandel Scientific, Germany). Statistics were applied in *n* = 13 rats. For the comparison of the effects of DCS (anodal and cathodal) on the rising slope_1–6_ for gastrocnemius muscle and tibialis anterior muscle on both sides, we used the repeated measure analysis of variance, followed by the Holm-Sidak test. To compare the effects of DCS (anodal versus cathodal) on the amplitudes of corticomotor responses on each side, we applied the Friedman repeated measures analysis of variance on ranks, followed by the Tukey test for multiple comparison procedures. For the comparison of the values of NP50 between the 3 experimental conditions (baseline, post-ADCS, and post-CDCS) in left gastrocnemius muscle, we used the repeated measures analysis of variance, followed by the Holm-Sidak test. For the values of NP50 in left tibialis anterior muscle, we used the repeated measures analysis of variance on ranks, followed by the Tukey test. To assess the effects of DCS on afferent inhibition, we applied a one-way analysis of variance, followed by the Holm-Sidak method or the Friedman repeated measures analysis of variance on ranks according to the results of normality assessment. To compare the ratios mean *F*/mean *M* responses, as well as the persistence of *F* wave on each side in the 3 conditions (basal, postanodal stimulation, and postcathodal stimulation), we applied the Friedman repeated measures analysis of variance on ranks, followed by a Tukey test for multiple comparison procedures. To evaluate the effects of DCS on the ratios *H*_max⁡_/*M*_max⁡_ on each side at 2 times *M* threshold and 3 times *M* threshold, we applied the one-way analysis of variance if normality test passed or the Friedman repeated measures analysis of variance on ranks if normality test failed. *P* values lower than 0.05 were considered as statistically significant.

## 3. Results

We did not observe macroscopical lesions in cerebellar hemispheres, in motor, or premotor areas. In particular, we did not find any visible lesion under the DCS electrodes.

### 3.1. Corticomuscular Responses and Afferent Inhibition

Latencies of corticomotor responses were between 7.6 and 9.4 msec in both sides. CMAPs of the gastrocnemius muscles were similar on the left side and right side, as expected in the absence of peripheral nerve injury (range of peak-to-peak intensities: 6.6 to 12.9 mV).

**Figure 1:**
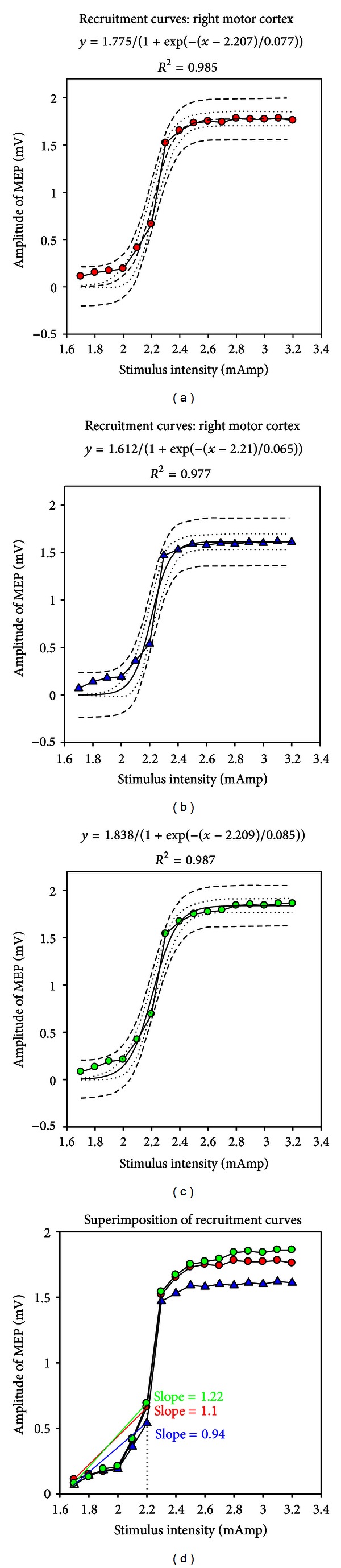
Examples of recruitment curves of corticomotor evoked responses (MEP: motor evoked responses) following incremental electrical stimulation of the right motor cortex for one rat. Recordings in left gastrocnemius muscle. (a) Recordings at baseline. (b) Recordings following application of anodal direct current stimulation over left cerebellar hemisphere. (c) Recordings following stimulation of left cerebellar hemisphere with cathodal direct current stimulation. (d) Superimposition of the 3 recruitment curves. Individual values are shown with red circles in (a), blue triangles in (b), and green circles in (c). Sigmoidal fitting (3 parameters: *y* = *a*/[1 + exp⁡(−(*x* − *x*_0_)/*b*)]); 95% prediction bands (dash) and 95% confidence bands (dotted) are shown in (a), (b), and (c). In (d), the slope from the first to the sixth point is smaller following anodal stimulation: 0.94 as compared to 1.1 at baseline and 1.22 after cathodal stimulation. Stimulation intensity is increased by steps of 0.1 mAmp following determination of the motor threshold on each side. Amplitudes are expressed in mV.

**Figure 2:**
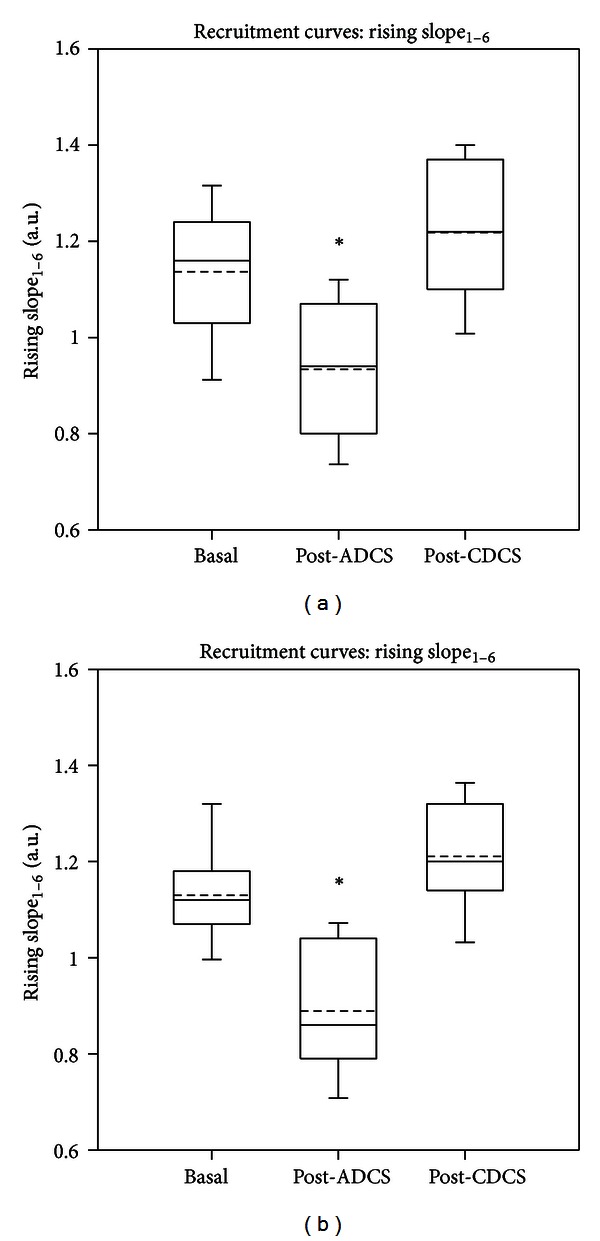
Box and whisker plots showing the effects of direct current stimulation applied over left cerebellar hemisphere on the slope between the first and the sixth points (slope_1–6_) of the recruitment curve for left gastrocnemius muscle (a) and for left tibialis anterior muscle (b). Basal: values in basal condition; post-ADCS: values obtained after application of anodal direct current stimulation over the dura; post-CDCS: values obtained after application of cathodal direct current stimulation over the dura. Medians (continuous lines) and means (dashed lines) are illustrated, as well as outliers (corresponding to the 5th and 95th percentiles). Values at baseline and values post-CDCS are significantly higher as compared to the values obtained post-ADCS. *P < 0.05 (*n* = 13 rats).

An example of the sigmoidal fitting of the recruitment curves of MEP recorded in left gastrocnemius muscle at baseline after cerebellar ADCS and after cerebellar CDCS is illustrated in Figure 1 (in (a), (b), and (c), resp.) for one rat. Both ADCS and CDCS preserved the sigmoidal shape of the recruitment curve, but ADCS reduced the maximal value of the fit, reducing the amplitudes of MEP, unlike CDCS. This was not the sole effect of ADCS. Indeed, anodal stimulation reduced the amplitudes of MEP in the critical phase of modulation of the recruitment curve. For *x* = *x*_0_, the *y* value (corresponding to 50% of the maximal value in the *y*-axis) was 0.8875 mV, 0.806 mV, and 0.919 mV, for baseline, after ADCS, and after CDCS, respectively. Moreover, the sixth point of the recruitment curve (corresponding to *x* = 2.2 mAmp) had a smaller value after ADCS. This is confirmed by the slope_1–6_ (see Figure 1(d)). In left gastrocnemius muscle, the analysis of variance in the group of 13 rats confirmed a significant intergroup difference for the slope_1–6_ between basal values, post-ADCS, and post-CDCS values (*P* < 0.001; Figure 2(a)). Pairwise multiple comparison procedure showed that values after ADCS were significantly smaller as compared to basal and post-CDCS values (*P* < 0.05). Although values after CDCS were slightly greater as compared to baseline values, the difference was not statistically different (*P* = 0.129). Very similar observations were made for the effects of anodal and cathodal stimulation on the slope_1–6_ of left tibialis anterior muscle (Figure 2(b)). Again, a highly significant intergroup difference (*P* < 0.001) was found. Values after ADCS were significantly smaller as compared to baseline and after CDCS (*P* < 0.05). The intergroup difference between baseline values and post-CDCS did not reach significance (*P* = 0.105). On the right gastrocnemius muscle (stimulation of the left motor cortex), application of DCS over left cerebellum (anodal or cathodal) did not change the features of recruitment curves. The maximal values of the fit remained unchanged, and the amplitudes of MEP at the critical phase of modulation of the recruitment curve (*y* for *x* = *x*_0_, and the values of the sixth points) remained unaffected. There was no intergroup difference for the slope_1–6_ between basal, post-ADCS, and post-CDCS values for right gastrocnemius muscle (*P* = 0.497) and right tibialis anterior (*P* = 0.383).

**Figure 3:**
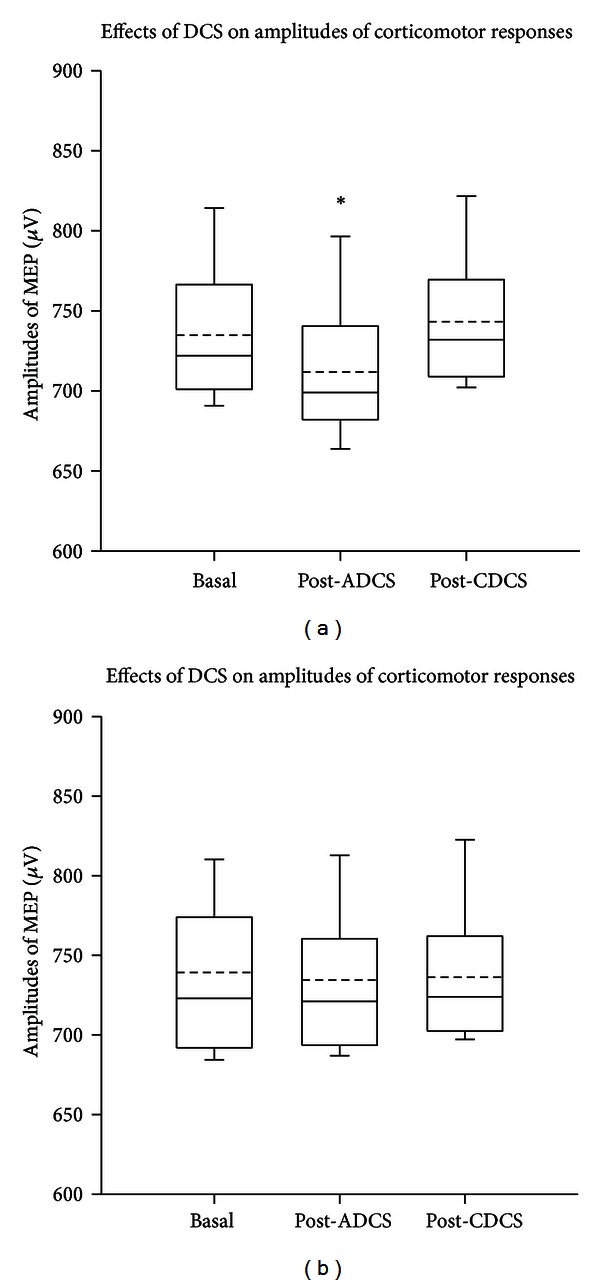
Box and whisker plots showing the effects of direct current stimulation applied over left cerebellar hemisphere on the amplitudes of corticomotor responses. Left and right panels correspond to values from left gastrocnemius muscle (a) and right gastrocnemius muscle (b), respectively. Basal: values in basal condition; post-ADCS: values obtained after application of anodal direct current stimulation over the dura; post-CDCS: values obtained after application of cathodal direct current stimulation over the dura. Medians (continuous lines) and means (dashed lines) are illustrated, as well as outliers (corresponding to the 5th and 95th percentiles). In left gastrocnemius muscle, values at baseline and values after CDCS are significantly higher as compared to the values obtained after ADCS. *P < 0.05 (*n* = 13 rats).

After ADCS, the amplitudes of corticomotor responses in left gastrocnemius muscle were significantly reduced as compared to basal values and values after CDCS (Figure 3(a); Friedman repeated measures analysis of variance on ranks: *P* < 0.001). Tukey test confirmed the depression of the magnitudes of motor responses induced by ADCS (*P* < 0.05). By contrast, values of corticomotor responses remained unchanged on the right gastrocnemius muscle (Figure 3(b); Friedman test: *P* = 0.232). Similar observations were made for left tibialis anterior muscle.

**Figure 4:**
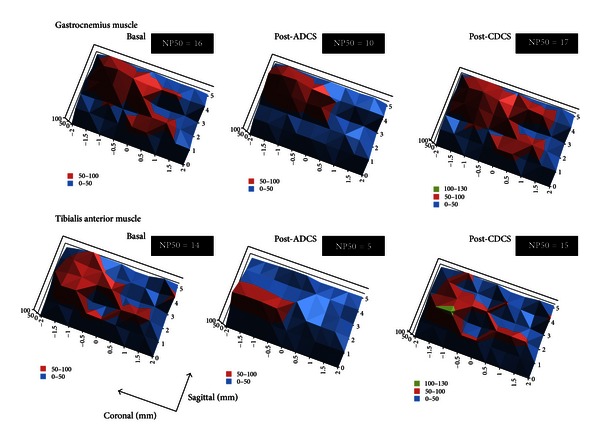
Motor maps corresponding to responses recorded in left gastrocnemius muscle (upper panels) and left tibialis anterior muscle (bottom panels), ipsilaterally to the site of cerebellar direct current stimulation for one rat. Data are represented using contour plots with *XYZ* coordinates. *x*-axis and *y*-axis refer to sagittal and coronal coordinates, respectively (coordinates of Bregma: 0/0 mm). Intensities of corticomotor responses are illustrated in *z*-axis using a linear scale. The so-called “hot spots” (shown in red) are identified. Stimuli are applied every mm in the sagittal axis and every 0.5 mm in the coronal axis (matrix of 6 × 9 = 54 sites of stimulation). Coordinates of stimulation are established using the stereotactic frame. Left panels: recordings in basal condition; middle panels: recordings after application of anodal direct current stimulation (post-ADCS); right panels: recordings after application of cathodal direct current stimulation (post-CDCS). Maximal responses obtained at baseline are set at 100%, for both the gastrocnemius muscle and tibialis anterior muscle. ADCS induces a reorganization of the corticomotor map, restricting the representation of the most intense motor responses around the hot spot (“focusing effect”) and reducing the intensity of the hot spot. By contrast, application of CDCS redistributes the representation of the most intense motor responses and increases the magnitude of the hot spot. The number of peaks in the *Z* axis with values above 50% of maximal corticomotor responses (NP50) is indicated in the upper right corner of each panel. Note the clear drop after ADCS and the slight increase after CDCS, as compared to baseline.

Figure 4 shows the corticomotor maps using contour plots with a linear representation of data along the *z*-axis, obtained on the left side for a couple of agonist/antagonist muscles. The so-called “hot spots” were clearly identified at baseline for both the gastrocnemius muscle (upper left panel) and the tibialis anterior muscle (lower left panel). Interestingly, ADCS induced a “focusing effect” in terms of representation of the motor maps for both muscles (middle panels): the highest motor responses were centered around the hot spot. CDCS reverted the effect (right panels). After ADCS, the value of NP50 dropped from 16 to 10 and from 14 to 5, for the gastrocnemius and tibialis anterior muscle, respectively. For the left gastrocnemius muscle, the mean number (±SD) of NP50 for the group of 13 rats was 16.92 ± 1.11, 10.23 ± 1.16, and 20.77 ± 1.64, respectively, in the basal condition, after ADCS, and after CDCS. The difference was statistically different (*P* < 0.001) between the 3 conditions as confirmed by the repeated measures analysis of variance. Pairwise comparison procedure with the Holm-Sidak test showed that the NP50 was significantly lower after ADCS as compared to the 2 other conditions (*P* < 0.05). In addition, the NP50 was significantly higher for CDCS as compared to basal values (*P* < 0.05). For the left tibialis anterior muscle, the repeated measures analysis of variance on ranks followed by the Tukey test showed statistically lower NP50 (*P* < 0.001) after ADCS (5.31 ± 0.94) as compared to the basal condition (14.84 ± 0.89) and after CDCS (14.30 ± 0.75). For left motor cortex, no change of the motor maps (related to right gastrocnemius muscle/right tibialis anterior muscle) was observed after application of DCS (anodal or cathodal) over left cerebellar hemisphere.

**Figure 5:**
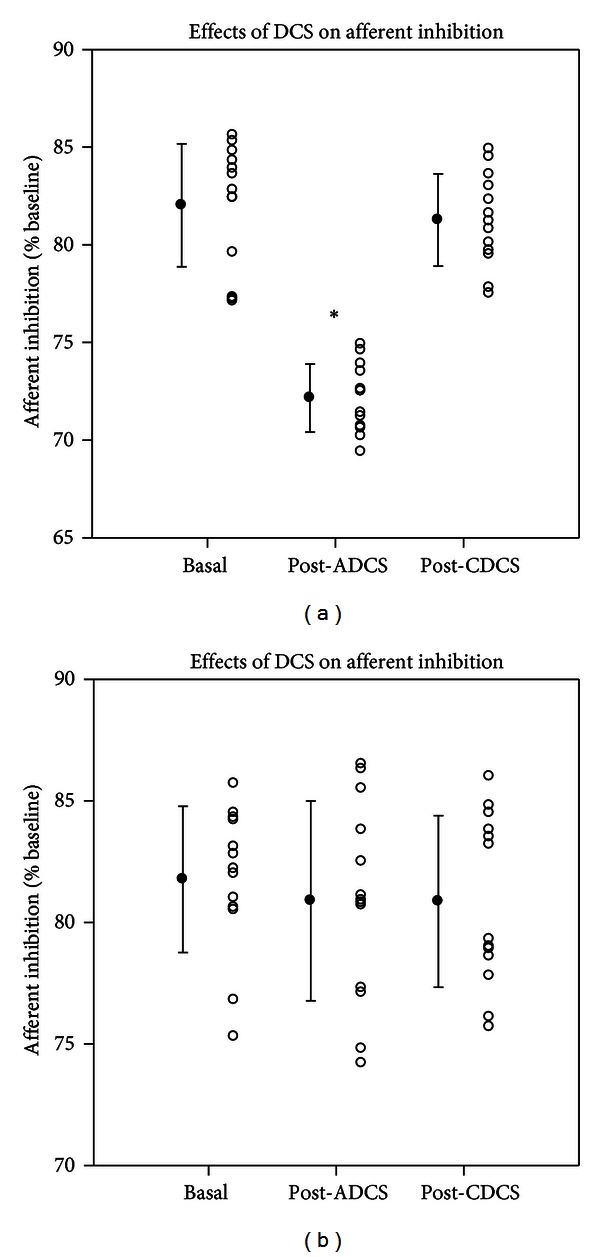
Effects of direct current stimulation (DCS) on afferent inhibition in left gastrocnemius muscle (a) and right gastrocnemius muscle (b) at baseline, after application of anodal DCS (ADCS) and cathodal DCS (CDCS) over left cerebellar hemisphere. A conditioning stimulus is applied on the sciatic nerve contralaterally to the stimulated motor cortex (right motor cortex and left motor cortex for (a) and (b), resp.) with an interstimulus interval of 45 msec. The afferent inhibition is expressed in % of baseline motor responses (obtained without conditioning stimulus). Mean values of motor evoked responses obtained in the baseline condition are set at 100% for each motor cortex. Mean values (filled circles; ±SD) as well as individual values (open circles) are shown. Cerebellar ADCS enhances the afferent inhibition for the ipsilateral muscle. *P < 0.05 (*n* = 13 rats).

In left gastrocnemius muscle, ADCS increased significantly the afferent inhibition as compared to baseline (Figure 5(a); Holm-Sidak test: *P* = 0.017). Values after CDCS were significantly higher as compared to the post-ADCS condition (*P* = 0.025) but remained similar to baseline measurements. In right gastrocnemius muscle, no DCS effect was found (Figure 5(b); *P* = 0.2).

### 3.2. *F* Waves and *H* Reflex

**Figure 6:**
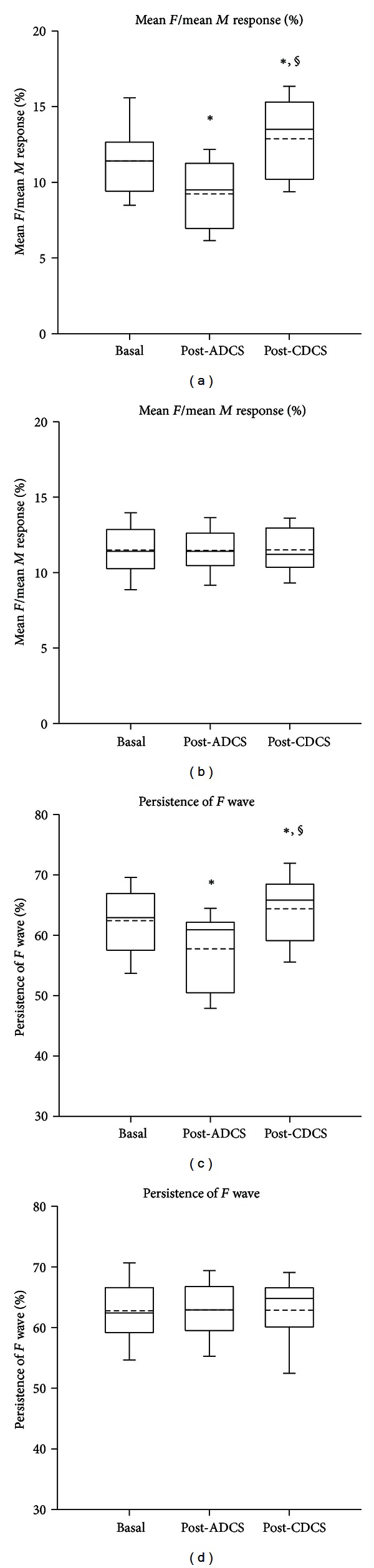
Box and whisker plots showing the changes in the excitability of the anterior horn of the spinal cord, assessed by the ratios mean *F*/mean *M* (expressed in %; (a, b)) and the persistence of the *F* wave (expressed in %; (c, d)) in *n* = 13 rats. Recordings in left gastrocnemius muscle (a, c) and right gastrocnemius muscle (b, d). Basal: values in basal condition; post-ADCS: values obtained after application of anodal direct current stimulation over left cerebellar hemisphere; post-CDCS: values obtained after application of cathodal direct current stimulation over left cerebellar hemisphere. Medians (continuous lines) and means (dashed lines) are illustrated, as well as outliers (corresponding to the 5th and 95th percentiles). In left gastrocnemius muscle, values after ADCS and values after CDCS are significantly lower and higher as compared to the basal values, respectively (*P < 0.05). Moreover, values after CDCS are significantly higher as compared to the values after ADCS (^§^ *P* < 0.05).

The *M* wave was of short latency (2.5 to 3.4 msec) [19]. As expected, *M* responses were unchanged by cerebellar DCS.  Figure 6 illustrates the ratios mean *F*/mean *M* responses and the persistence of *F* waves on left side (a, c) and on right side (b, d) for the gastrocnemius muscle. In left gastrocnemius muscle, a significant DCS effect was confirmed by the Friedman test (*P* < 0.001). The ratio mean *F*/mean *M* was depressed after ADCS as compared to baseline (Tukey test: *P* < 0.05), unlike the ratio mean *F*/mean *M* after CDCS which increased as compared to basal values (*P* < 0.05). On the right side, no DCS effect was observed (*P* = 0.926). Regarding the persistence of *F* wave, similar observations were made. On the left side, the DCS effect was highly significant (*P* < 0.001). Values after ADCS and after CDCS were significantly smaller and larger as compared to baseline values, respectively (Tukey test: *P* < 0.05 in both cases). No difference was found on the right side (DCS effect: *P* = 0.368).

**Figure 7:**
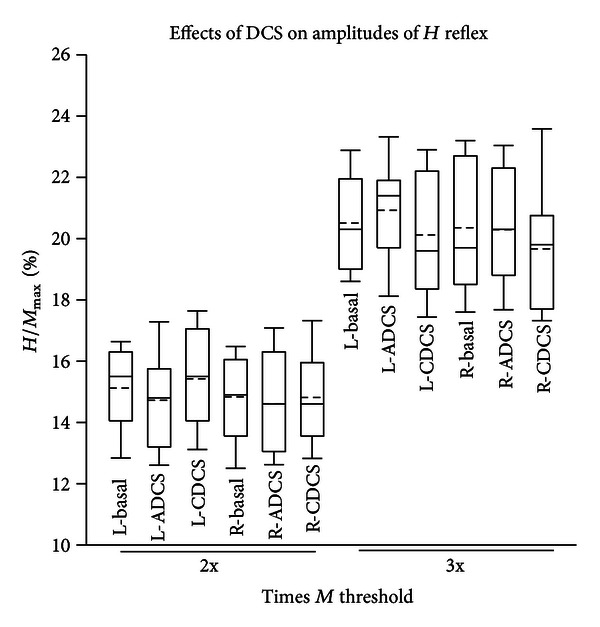
Box and whisker plots showing the *H*_max⁡_/*M*_max⁡_ ratios (expressed in %) at 2 times (2x) and 3 times (3x) the *M* threshold, in left (L-) and right (R-) plantaris muscles. Basal: values in basal condition; ADCS: values obtained after application of anodal direct current stimulation over left cerebellar hemisphere; CDCS: values obtained after application of cathodal direct current stimulation over left cerebellar hemisphere. Medians (continuous lines) and means (dashed lines) are illustrated, as well as outliers (corresponding to the 5th and 95th percentiles). No significant difference is observed (*n* = 13 rats).

The latency of the *H* reflex ranged from 10.3 to 12.6 msec. The *H*_max⁡_/*M*_max⁡_ ratios were found to be around 20%, as reported previously [3, 19]. The *H* reflex amplitudes increased as a function of stimulus intensity. Application of trains of DCS did not modify *H* reflex amplitudes. The threshold, the slope, and the *H*_max⁡_/*M*_max⁡_ ratio were similar before and after ADCS or CDCS. On the left side, no DCS effect was found at 2 times *M* threshold (*P* = 0.689) or at 3 times *M* threshold (*P* = 0.3), as illustrated in Figure 7. On the right side, no DCS effect was found either (values at 2 times *M* threshold: *P* = 0.905; values at 3 times *M* threshold: *P* = 0.454).

## 4. Discussion

Our experiments show that trains of cerebellar DCS applied on the dura modulate in a more powerful way than one might expect the excitability of the contralateral motor cortex, since the representation of corticomotor maps was modified. We investigated specifically the aftereffects of DCS rather than intra-DCS effects, because the anodal stimulation-elicited intra-DCS effects are far less prominent as compared to the aftereffects of DCS, while for CDCS the intra- and post-DCS effects are relatively similar [23]. We have applied an intermittent pattern of stimulation at relatively high stimulation intensities [4]. No macroscopical lesion was identified under the DCS electrodes. One interesting advantage of anodal DCS applied over the cerebellum is its capacity to tune the excitability of the spinal cord, unlike transcranial magnetic stimulation (TMS) or trains of DCS applied on the motor cortex [4]. Another potential advantage of the technique of epidural or surface electrical stimulation is the more focal stimulation in terms of spatial resolution as compared to TMS. Furthermore, TMS carries a higher risk for seizure occurrence, especially in patients with lesions of the cerebral cortex [24].

Two differences with published human studies must be noted. First, the experiments reported here were performed by carefully exposing the dura in order to obtain a high degree of spatial resolution for stimulation, unlike the human experiments applying the constant current directly over the scalp. However, recent observations have confirmed that stimulation of the cerebellum by removing the soft tissues but keeping the skull intact is also associated with shape tuning of corticomotor maps (unpublished data). Second, our experiments are performed under general anesthesia. This is required to obtain stable corticomotor maps but has the obvious disadvantage of sedation which interferes with the activity of brain networks controlling limb movements. However, performing detailed and reproducible corticomotor maps in awake rats is still a challenge, despite the development of epidurally implanted thin-film microelectrode arrays [16]. A recent study in alert behaving rabbits has elegantly demonstrated the feasibility of investigating the mechanisms of associative learning using DCS applied over the somatosensory cortex, showing the neuromodulation of a complex cortical process with DCS [9].

Anodal epidural DCS of the cerebellum appears to be a very attractive tool to modulate and even shape the corticomotor representation of limb muscles. Indeed, not only does anodal epidural DCS decrease the magnitude of corticomotor responses and enhance the afferent inhibition process associated with peripheral stimuli, but it also changes the representation pattern of limb muscles over the motor cortex. We observed a “focusing effect” around the hot spot, characterized by a concentration of the highest motor responses around the hot spot. An opposite effect was observed with cathodal epidural DCS, as confirmed by the changes of the parameter NP50. In terms of excitability of the spinal cord, anodal epidural DCS exerted reverse effects as compared to cathodal epidural DCS at the level of the anterior horn, whereas the monosynaptic reflexes remained unchanged by both techniques.

Cerebellar information is funnelled to the primary motor cortex via the ventrolateral thalamic group which projects mainly to cortical layers IV and V [25]. Through this channel, inputs can modulate the efficacy of the interconnections among cortical neurons, adjusting the circuitry of the motor cortex in various contexts. Cerebellar predictions and updates based on sensory events would be possible, thanks to the numerous projections received by the cerebellum, the huge computing capacities of the cerebellar circuitry, and the interactions between mossy and climbing fibres [26]. It is established that single pulse TMS over the cerebellum changes contralateral motor cortex excitability, by a mechanism of cerebellocortical inhibition [27, 28]. In human, high-voltage conditioning electrical stimuli over the lateral cerebellar hemisphere preceding a magnetic stimulus (by 5–7 msec) delivered contralaterally on the motor cortex depress EMG responses, as a result of a stimulation of the cerebellar cortex, causing an inhibition of the dentatothalamocortical pathway [28, 29]. This inhibitory effect is absent in cerebellar patients when lesions involve dentate nuclei or the superior cerebellar peduncle [27]. Hemicerebellectomy in human causes a total loss of the inhibitory effect [30]. The phenomenon has been confirmed in hemicerebellar ablation in rats. One Hz repetitive TMS (rTMS) over the cerebellar cortex increases intracortical facilitation at the level of the motor cortex contralaterally, and low-frequency cerebellar rTMS trains affect motor intracortical excitability beyond the duration of the train [31]. Our results obtained with anodal DCS can be interpreted in terms of disfacilitation of the dentatothalamocortical pathway: anodal DCS would enhance the inhibition exerted by the cerebellar cortex over cerebellar nuclei, thus, removing the facilitatory cerebellofugal drive exerted by cerebellar nuclei. By contrast, cathodal cerebellar DCS would decrease the inhibition of cerebellar cortex over nuclei, although a purely opposite effect between anodal and cathodal techniques cannot be put forward. Recent human studies have provided a strong support to the concept that transcranial DCS of the cerebellum modulates in a focal and polarity-specific manner cerebellar excitability, very likely by acting on populations of Purkinje cells in the cerebellar cortex [8].

We have demonstrated in a previous study that (1) high-frequency stimulation in the interpositus nucleus at low intensities decreases the amplitudes of corticomotor responses recorded ipsilaterally, suggesting a decreased excitability of the pyramidal system, and (2) anodal DCS of the motor cortex reverts this depressive effect [4]. The efferent pathways from cerebellar nuclei tune the excitability of segmental motoneurons via ascending and descending pathways, leading to decreased excitability of the corticomotor system in case of hemicerebellar ablation [32]. The specific effects of cerebellar nuclei stimuli on the excitability of lumbar alpha motoneurons are dependent upon the location within the nuclei at which the stimuli are administered. Conditioning trains of dentate nucleus stimuli change the postsynaptic potentials evoked in motoneurons by stimulation of group Ia/Ib afferents in appropriate peripheral nerves. Experimental data support the existence of an excitatory cerebello-thalamo-cortico-spinal pathway which affects the excitability of motoneurons. Indeed, when a cooling of the motor cortex is applied, the procedure cancels an excitatory component from the intracellularly recorded response evoked in lumbar motoneurons by dentate stimulation [32]. In addition, descending pathways from the brainstem contribute to the control of muscle tension associated with limb movements. Functional blockade of cerebellar interpositus nucleus with tetrodotoxin reduces the slope of the *H* reflex recruitment curve without affecting the *H*_max⁡_/*M*_max⁡_ ratios and depresses both *F* wave persistence and mean *F*/mean *M* ratios [3]. The potential importance of these nucleospinal projections in the understanding of cerebellar deficits might have been neglected in the past.

The use of cerebellar stimulation to modulate motor function was studied by Cooper about 4 decades ago [33]. This author found that cerebellar stimulation reduces the amplitudes of somatosensory evoked responses. Disorders such as spasticity, or even epilepsy, have been considered as pathological conditions which could benefit from cerebellar stimulation. More recently, DCS has been shown to improve motor or cognitive performance in disorders affecting the supratentorial regions [10, 12]. By facilitating synaptic connectivity, DCS may affect motor skill acquisition and might promote or enhance recovery following a stroke. A beneficial effect of anodal DCS has been shown on working memory [12]. Intermittent pattern of stimulation (15 seconds on/15 seconds off over periods of 30 minutes) improves sleep-dependent consolidation of memory [34]. Overall, many neurological disorders in which a manipulation of cortical excitability might be beneficial—for instance, to promote the plastic changes underlying learning and recovery—are potential therapeutic targets for DCS. Gait deficits, whose incidence and prevalence are high especially in cerebellar disorders, are now considered as one of them: cerebellar anodal DCS applied during locomotor adaptation improves the adaptive process, whereas cathodal cerebellar DCS slows it down [7]. The technique might also have a diagnostic application to detect starting lesions of the motor cortex, the absence of sustained excitability change being indicative of damaged corticospinal cells [35]. Recent advances in our understanding of the roles of the cerebellum in cognition and emotion highlight the importance of reconsidering analysis of the cerebellocerebral projections [36]. For instance, the fact that cathodal cerebellar DCS impairs verbal working memory without impairing word reading opens novel doors both for research and therapies [37].

In conclusion, our findings show that DCS applied over the cerebellum exerts a potent modulatory effect upon the activity of the motor cortex/anterior horn of the spinal cord. This opens the question of future applications aiming to speed up learning and adaptative processes associated with motor behaviour, with the final goal of improving performance [6, 38]. Cerebellum as a primary target of neurostimulation has received relatively little attention as compared to primary motor cortex and premotor cortex [38]. An interesting field of research deserving further works is animal and human ataxic disorders for which the excitability of the motor cortex/spinal cord is impaired. Disorders characterized by a decreased inhibitory drive of the cerebellar cortex over cerebellar nuclei, such as cerebellar cortical atrophy affecting primarily the cerebellar cortex, represent a potential therapeutic target of anodal cerebellar DCS, whereas disorders in which underactivity of cerebellar nuclei is suspected, such as a stroke, could be managed with cathodal cerebellar DCS. With a better understanding of the effects of DCS upon cerebellar circuitry, the field of cerebellar neuromodulation has entered in a resurgence stage. Attempts to modulate neuronal activities by applying weak electric currents transcranially are certainly not novel [39, 40], but they are now restarting intensely with fresh views and novel findings, including those in the field of ataxiology.

## Supplementary Material

Supplementary Material: Illustration of the "focusing effect" exerted by AtDCS on the motor maps of both the gastrocnemius muscle and the tibialis anterior muscle, using a natural log scale in the Z axis (minimal and maximal values along the Z axis are identical to values shown in Figure 3). Crossing of dotted lines correspond to the maximal corticomotor responses. Colors (green, orange and red) assigned to the ranges of obtained values. Green: intensity in the range I < 20; Orange: intensity in the range 20 < I < 54; Red: intensity in the range 54 < I < 120.
