# Supplementary material for: Trains of Epidural DC Stimulation of the Cerebellum Tune Corticomotor Excitability
Source: Neural Plast. 2013 May 20;2013:613197. doi: 10.1155/2013/613197 (PMC3673402; doi:10.1155/2013/613197)

Supplement: illustration of the “focusing effect” exerted by AtDCS on the motor maps of both the gastrocnemius muscle and the tibialis anterior muscle, using a natural log scale in the Z axis (minimal and maximal values along the Z axis are identical to values shown in Figure 3). Crossing of dotted lines correspond to the maximal corticomotor responses. Colors (green, orange and red) assigned to the ranges of obtained values. Green: intensity in the range I < 20; Orange: intensity in the range 20 < I < 54; Red: intensity in the range 54 < I < 120.


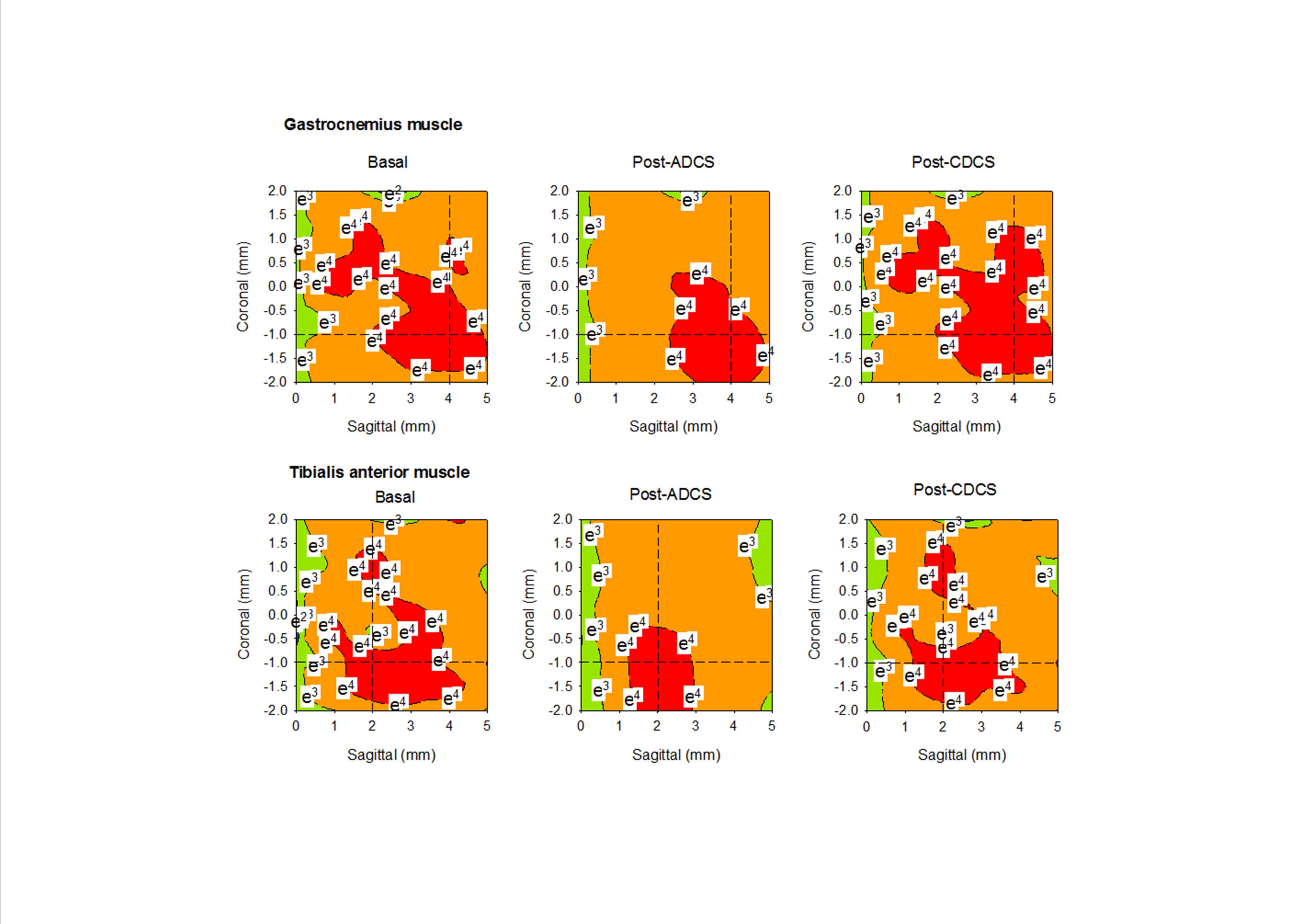

Supplement: Supplementary file 1 — Supplementary Material: Illustration of the "focusing effect" exerted by AtDCS on the motor maps of both the gastrocnemius muscle and the tibialis anterior muscle, using a natural log scale in the Z axis (minimal and maximal values along the Z axis are identical to values shown in Figure 3). Crossing of dotted lines correspond to the maximal corticomotor responses. Colors (green, orange and red) assigned to the ranges of obtained values. Green: intensity in the range I < 20; Orange: intensity in the range 20 < I < 54; Red: intensity in the range 54 < I < 120. [file 613197.f1.docx]
